# Conversion of ER, PR, HER2 and Ki-67 and Prognosis in breast cancer metastases to the brain

**DOI:** 10.3389/fneur.2022.1002173

**Published:** 2022-10-24

**Authors:** Chen Jiaxin, Zhou Jinmei, Zhang Huiqiang, Wu Xuexue, Wang Xiaobo, Zhang Shaohua, Tai Yanhong, Jiang Zefei, Wang Tao

**Affiliations:** ^1^Department of Oncology, The Fifth Medical Center of Chinese PLA General Hospital/Chinese PLA Medical School, Beijing, China; ^2^Department of Oncology, The Fifth Medical Center of Chinese PLA General Hospital, Beijing, China; ^3^Anhui Medical University, Hefei, China; ^4^Southern Medical University, Guangzhou, China

**Keywords:** breast cancer, brain metastases, ER, PR, HER-2

## Abstract

**Objective:**

This study aimed to analyze the expression levels of estrogen receptor (ER), progesterone receptor (PR), human epidermal growth factor receptor 2 (HER-2), and Ki-67 proliferation index in the brain metastatic lesions and primary lesions in Chinese patients with breast cancer brain metastasis (BCBM) and determine the correlation between their changes and patients' survival.

**Methods:**

A retrospective analysis was performed on patients with BCBM. The clinical characteristic of these patients was collected. The differences in the expression levels of the ER, PR, HER-2, and Ki-67 index between the primary lesions and brain lesions were evaluated, and the association between the differences and survival was analyzed.

**Results:**

The conversion rate of anyone receptor (ER, PR, or HER2) between the primary lesions and brain metastatic lesions was 45.0% (18/40), of which the ER inconsistency rate was 25.0%, the PR inconsistency rate was 22.5%, and the HER-2 inconsistency rate was 15.0%, and the receptor conversion resulted in a subtype conversion of 27.5% (11/40). The patients with HER-2 expression discordance between the primary lesions and the brain metastatic lesions had significantly longer survival times (58.9 vs. 26.4 months, *P* = 0.04) after diagnosis of brain metastases.

**Conclusion:**

In this study, 45.0% of breast cancer patients developed biomarker-conversion between the primary lesions and brain metastatic lesions, and the differences in the expression levels of the ER, PR, and HER-2, the change in Ki-67 index between the primary lesions and brain lesions may predict patients' survival.

## Introduction

There were 19.29 million new cancer cases worldwide in 2020 and breast cancer surpassed lung cancer as the most commonly diagnosed cancer, with an estimated 2.26 million new cases, followed by lung cancer (2.20 million) according to the latest global cancer statistics released by the International Agency for Research on Cancer (IARC) at the World Health Organization (WHO) in 2020 (1). Based on the latest data released by Chinese experts, it was estimated that there would be 429,105 new breast cancer cases in Chinese women in 2022 and breast cancer would be the most common cancer in females in China (2). Breast cancer has become the most common malignant tumor, seriously threatening the health of Chinese women.

With an improvement in the treatment of breast cancer in recent years, the 5-year survival rate of patients with early-stage breast cancer exceeded 90%, but 30–40% of patients still experienced recurrence and metastasis, and the 5-year survival rate of breast cancer patients with distant metastases was only about 30% (3). Advances in systemic therapy improved the survival of breast cancer patients, but the incidence of brain metastasis also increased. Brain metastases occurred in up to 40–50% of patients with HER2-positive and triple-negative breast cancer and 14% of patients with hormone receptor (HR)-positive breast cancer (4–7). Furthermore, brain metastasis was an important risk factor, threatening patients' survival, and the 1-year survival rate of patients with brain metastasis was only about 20% (8).

The current treatment of brain metastasis is still mainly based on local therapies, which include surgery, stereotactic radiosurgery, and whole-brain radiation therapy (WBRT). Drug therapy showed good clinical efficacy in patients with HER2-positive breast cancer, but effective drug therapy in HR-positive and triple-negative breast cancer patients with brain metastases was lacking (9). Due to limited treatment options, the average 1-year survival rate of patients with brain metastasis was only about 20% (10).Therefore, the treatment of brain metastasis is currently difficult in clinical practice, which requires more in-depth basic and clinical research to search for different treatment options in the future.

With a deep understanding of the molecular mechanism of breast cancer, the diagnosis and treatment of breast cancer entered the era of molecular typing and precision treatment, and the breast cancer was sub-grouped into luminal A, luminal B, HER-2, and triple-negative breast cancers (TNBC) based on the expression of ER, PR, HER-2, and the Ki-67 proliferation index. However, it is impossible to determine the receptor expression profiles of metastatic lesions due to limited access to brain lesions, which leads to the treatment of BCBM usually based on the receptor profiles of the primary lesions. However, the receptor expression profiles between primary lesions and brain metastatic lesions were not completely consistent, and more and more studies confirmed that conversion of the receptor involvement between primary lesions and metastatic lesions frequently occurred during the progression of breast cancer (11, 12). A previous review showed that ER, PR, and HER-2 expression discordance rates in primary and metastatic lesions were 6–40, 21–41, and 1–43%, respectively (13). Furthermore, the discordance rates of ER, PR, and HER2 expression ranged from 8 to 23% in a meta-analysis that included 48 studies (14).

This inconsistency appears to occur across all metastatic lesions and may require subtype-oriented treatment strategies. Current guidelines for the management of breast cancer recommend re-biopsy and reassessment of ER, PR, and HER-2 status in patients with distant metastases (9, 15, 16). However, our understanding of receptor expression in BCBM was limited due to the aggressive nature of neurosurgery and the resection or biopsy is currently not a standard treatment option for most BCBM patients. Although minimally invasive diagnostic methods targeting the central nervous system such as liquid biopsies or blood tests were emerging (17), these methods were largely limited to the research studies. Several studies investigated the inconsistency in BCBM, but most studies had relatively small sample sizes that caused the inconsistency in patients' prognosis, and hence, treatment decisions remained challenging (18–21). Therefore, more studies should be conducted to comprehensively understand the biomarker expression levels of intracranial lesions and their differences from extracranial lesions in BCBM patients, and the impact of such differences on prognosis and treatment options.

In this study, we aimed to analyze the expression levels of the ER, PR, HER-2, and Ki-67 in the primary lesions and brain lesions in Chinese breast cancer patients with brain metastases, the differences between the primary lesions and brain lesions, and the impact of the inconsistency on patients' prognosis, which might provide a new basis for the individualized treatment options.

## Methods

### Patients

This study retrospectively collected clinical data on patients who were diagnosed with breast cancer in the Department of Breast Oncology, the Fifth Medical Center of PLA General Hospital from January 1998 to November 2021, and these patients developed brain metastases during treatment or follow-up period (the follow-up time was from the diagnosis of primary breast cancer). All patients had neurosurgical resection of brain metastases. All patients whose ECOG ≤ 2 were enrolled in the study. The data were collected from breast cancer patients with brain metastases who had pathological profiles of both primary lesions and brain metastatic lesions. The collected data included the clinical information of the patients and the pathological profiles of the primary lesions, non-primary extracranial lesions, and brain metastatic lesions, and the general information such as the date of diagnosis of breast cancer, the histological type of primary tumor, the grade of breast cancer and the breast cancer stage at the time of diagnosis, the molecular typing, the immunohistochemical indicators, the subsequent date of diagnosis of BCBM, the number and location of metastatic sites at diagnosis, the number and location of BCBM lesions, the immunohistochemical indicators of BCBM lesions, and the overall survival of patients. All FFPE (Formalin-fixed paraffin-embedding) had a pathology review by two Pathologists.

### Imunohistochemistry

Immunohistochemistry (IHC) was performed to detect the status of ER and PR, and the cut-off value of positivity was set at 1%. HER-2 overexpression was defined as an immunohistochemical membrane staining with a score of 3+, and HER2-negative expression was divided into HER 2+ and 0-point score, and fluorescence *in situ* hybridization (FISH) was performed when the HER2 IHC score was ambiguous (2+). According to the American Society of Clinical Oncology/College of American Pathologists (ASCO/CAP) guideline recommendations for HER2 testing, it was judged as HER-2 positive if the ratio of HER-2/CEP17 was greater than or equal to 2.0 or the copy number of HER-2 gene was greater than or equal to 6. Immunohistochemistry (IHC) was performed to detect the ki-67index.

### Statistical analysis

Normally distributed continuous data were expressed as mean ± standard deviation and non-normally distributed continuous data are expressed as median (interquartile range). The differences between the groups were compared using chi-square or Fisher's exact test, and the univariate and multivariate logistic regression analyses were performed to determine the predictors of receptor expression inconsistency. log-rank test was used to conduct survival analyses. Subgroup analysis was performed based on important clinical factors and the Kaplan-Meier method was used to estimate the OS (Overall Survival, OS) and its 95% CIs. All statistics were performed using SPSS version 22.0 and R version 3.4.3 software. Two-sided statistical tests were used in this study and a *P*-value of < 0.05 were considered statistically significant.

All data and specimen testing retrievals were approved by the ethics committee of our hospital and all patients provided written informed consent.

## Results

A total of 40 breast cancer patients with brain metastases were included in the analyses of this study, whose ages ranged from 27 to 64 years (median age, 44.5 years). As of November 2021, the median follow-up time was 61.7 months, a total of 26 patients died, 6 (15.0%) patients had liver metastases, 14 (35.0%) patients had lung metastases, and 7 (17.5%) patients had bone metastases. The baseline characteristics are shown in Table 1.

**Table 1:** Baseline characteristics.

**Characteristics**	**Group**	**N**	**%**
Median age (years)	Value	44.5	
	Range	27–64	
Median DFS (months)	Value	17	
	Range	0–252	
Median Brain metastasis-free survival (months)	Value	29.3	
	Range	0–255.7	
Molecular typing	Luminal	7	17.5
	HER-2 +	24	60.0
	TNBC	9	22.5
TNM Stage			
	I	3	7.5
	II	20	50.0
	III	14	35.0
	IV	3	7.5
Number of brain metastases at diagnosis	Single	21	52.5
	Multiple	19	47.5
Meningeal metastases at diagnosis	Yes	4	10.0
	None	36	90.0
Symptoms at diagnosis of brain metastases	Yes	25	62.5
	None	15	37.5
Extracranial lesions			
	Lymph nodes	15	37.5
	Bone	7	17.5
	Liver	6	15.0
	Lung	14	35.0

The positive rates of ER, PR, and HER-2 in the primary lesions were 35.0% (14/40), 30.0% (12/40), and 60.0% (24/40), respectively, and the positive rates of ER, PR, and HER-2 in the brain metastatic lesions were 35.0% (14/40), 12.5% (5/40), and 55.0% (22/40), respectively. The immunohistochemical characteristics of the primary lesions and brain metastases are shown in Table 2.

**Table 2:** Summary of receptor conversion between the primary lesions and brain metastatic lesions.

**Brain metastatic lesions**	**Primary lesions**		**Total**
	**–**	**+**	
ER			
–	21 (52.5%)	5 (12.5%)	26 (65.0%)
+	5 (12.5%)	9 (22.5%)	14 (35.0%)
PR			
–	27 (67.5%)	8 (20.0%)	35 (87.5%)
+	1 (2.5%)	4 (10.0%)	5 (12.5%)
HER-2			
–	14 (35.0%)	4 (10.0%)	18 (45.0%)
+	2 (5.0%)	20 (50.0%)	22 (55.0%)

The conversion of receptor involvement between the primary lesions and metastatic lesions is shown in Table 3, and the inconsistency rate of ER, PR, and HER-2 expressions was 25.0% (10/40), 22.5% (9/40), and 15.0% (6/40), respectively.

**Table 3:** The receptor conversion trend between the primary lesions and brain metastatic lesions.

**Status of receptor**	**Positive-to-negative**	**Negative-to- positive**	**Total**
ER	5 (12.5%)	5 (12.5%)	10
PR	8 (20.0%)	1 (2.5%)	9
HER-2	4 (10.0%)	2 (5.0%)	6

**Figure 1:**
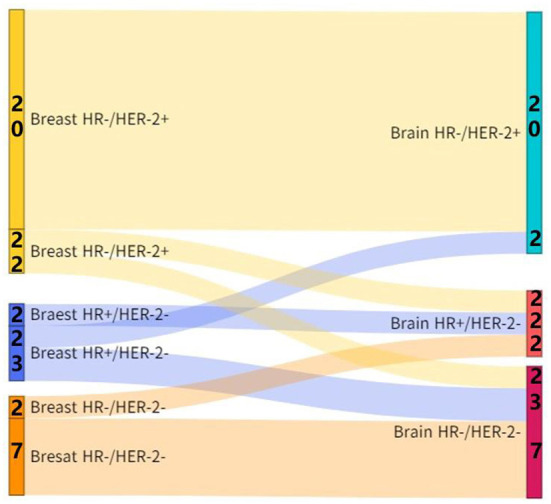
Subtype conversion between the brain metastatic lesions and primary lesions in patients with breast cancer.

The ratios of positive-to-negative and negative-to-positive in ER conversion were 12.5% (5/10) and 12.5% (5/10), respectively, and the ratios of positive-to-negative and negative-to-positive in PR conversion were 20.0% (8/9) and 2.5% (1/9), respectively, and the ratios of positive-to-negative and negative-to-positive in HER-2 conversion were 10.0% (4/6) and 5.0% (2/6), respectively. The inconsistency rate of the receptor (ER, PR, or HER2) between the primary lesions and the brain metastatic lesions was 45.0% (18/40). And the incidence of subtype conversion of breast cancer due to receptor inconsistency was 27.5% (11/40) (Figure 1).

**Figure 2:**
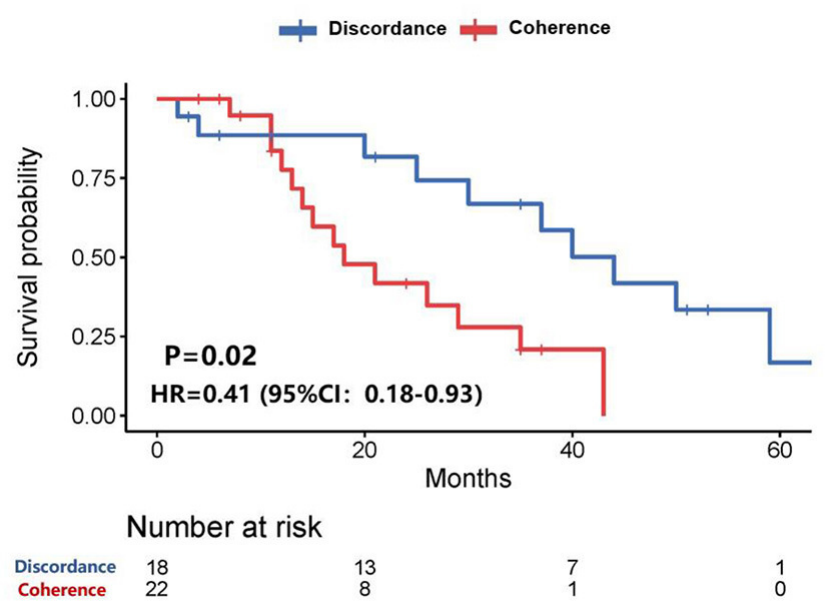
Survival curves of patients with consistent expression of anyone receptor vs. patients with inconsistent expression of anyone receptor after diagnosis of brain metastases.

Among all patients, who with any of the receptor (ER, PR, or HER-2) conversion between the primary lesions and brain metastatic lesions had a longer survival time than those patients without receptor conversion (39.6 vs. 18.3 months, *P* = 0.02) after diagnosis of brain metastases (Figure 2).

**Figure 3:**
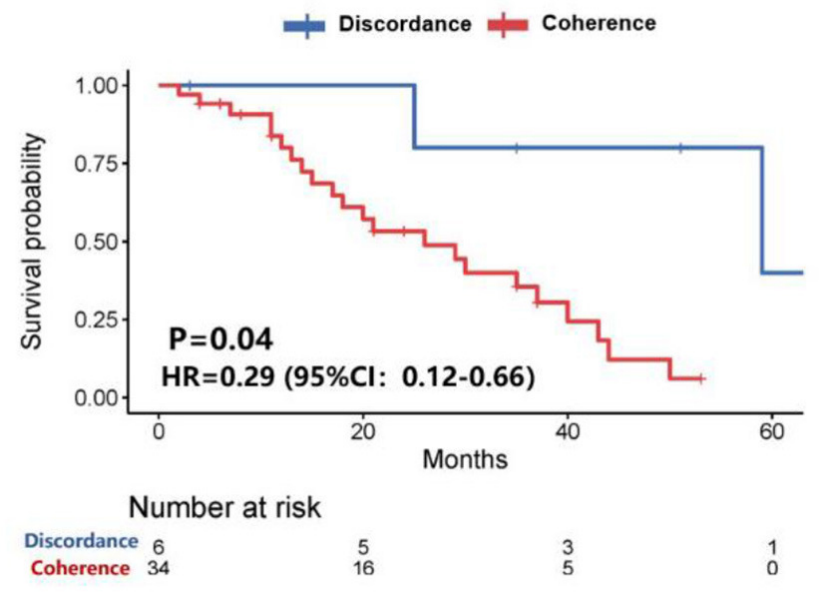
Survival curves of patients with consistent HER-2 expression vs. patients with inconsistent HER-2 expression after diagnosis of brain metastases.

**Figure 4:**
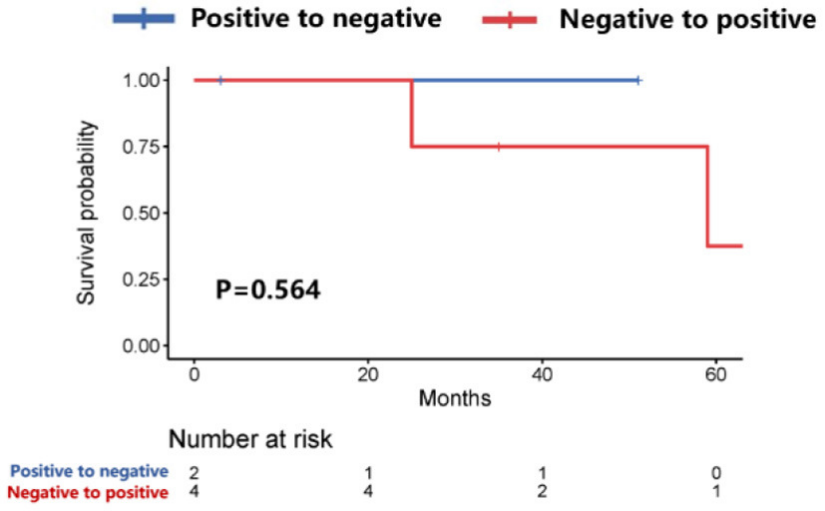
Survival curves of patients with positive-to-negative HER-2 expression vs. patients with negative-to-positive HER-2 expression after diagnosis of brain metastases.

Further analysis showed that patients with HER-2 conversion had a longer survival time (58.9 vs. 26.4 months, *P* = 0.04) after the diagnosis of brain metastases (Figure 3), of which patients whose HER-2 expression was converted from positive in the primary lesions to negative in the brain metastatic lesions were better than those patients whose HER-2 expression was converted from negative in the primary lesions to positive in the brain metastatic lesions (*P* = 0.173). However, the median survival time could not be evaluated due to the small sample size (Figure 4).

**Figure 5:**
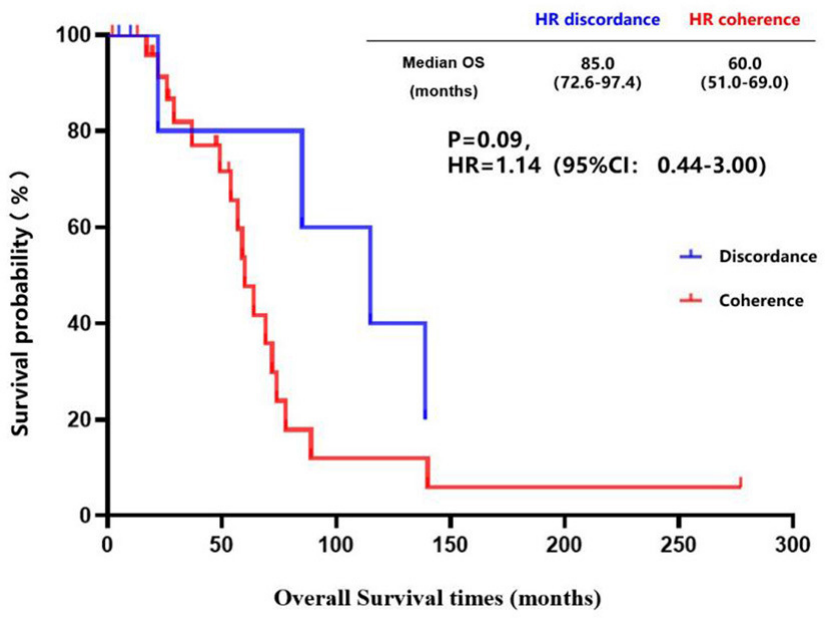
Kaplan-Meier curves of the overall survival in patients with consistent HR expression vs. patients with inconsistent HR expression.

**Figure 6:**
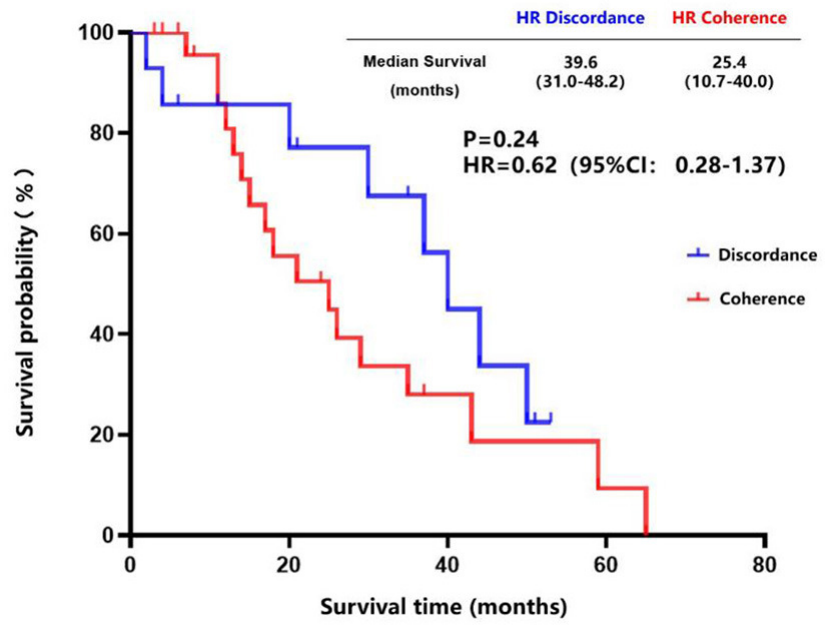
Survival curves of patients with consistent HR expression vs. patients with inconsistent HR expression after diagnosis of brain metastases.

The inconsistency analysis of the HR expression suggested that patients with HR conversion between the primary lesions and brain metastatic lesions had a longer OS than those patients without HR conversion, but no statistical difference was found (85.0 vs. 60.0 months, *P* = 0.09) (Figure 5). Also, patients with HR conversion had a longer survival time compared to those without HR conversion (39.0 vs. 25.4 months, *P* = 0.24) after diagnosis of brain metastases (Figure 6).

**Figure 7:**
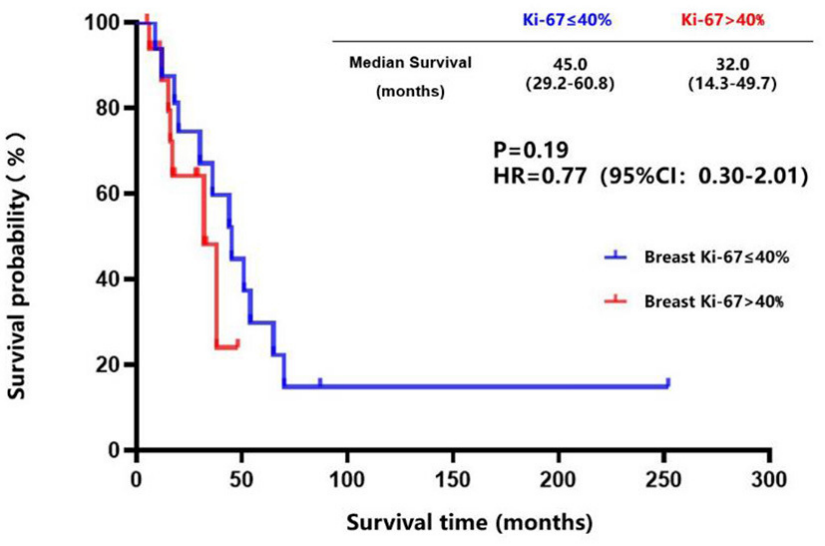
Kaplan-Meier curves of the metastasis-free survival in breast cancer patients with brain metastasis whose Ki-67 of ≤ 40% compared to those with Ki-67 of >40% in the primary lesions.

**Figure 8:**
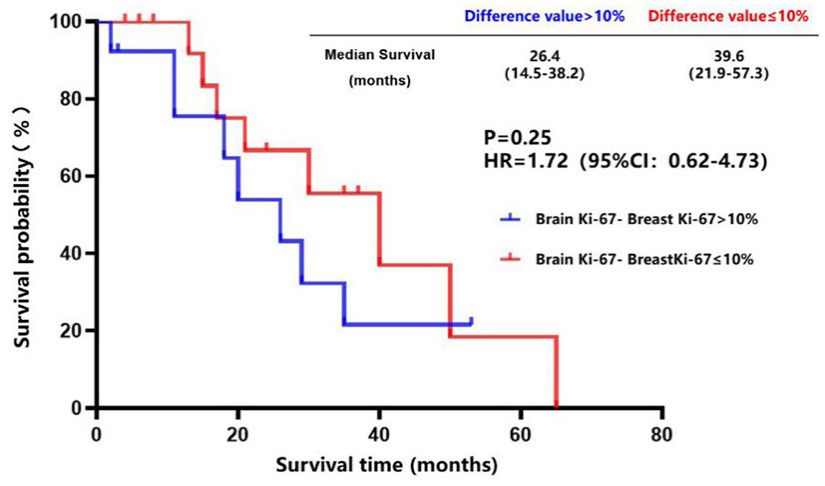
Survival curves of patients whose Ki-67 index difference of >10% vs. patients whose Ki-67 index difference of ≤ 10% after diagnosis of brain metastases.

The breast cancer patients with brain metastasis and Ki-67 of ≤ 40% in the primary lesions had a longer brain metastasis-free survival compared to those with Ki-67 of > 40% in the primary lesions (45.0 vs. 32.0 months, *P* = 0.19) (Figure 7), although no statistical difference was found. There was a trend toward better survival in patients whose Ki-67 index differences between the brain metastatic lesions and primary lesions were less than or equal to 10% compared to those patients whose Ki-67 index differences were more than 10% (39.6 vs. 26.4 months, *P* = 0.25) (Figure 8).

## Discussion

We retrospectively collected clinical data of 40 breast cancer patients with brain metastases for the analyses of this study and analyzed the incidence and clinical significance of conversion of receptor involvement between the primary lesions and brain metastatic lesions.

The proportion of patients with solitary brain metastases was higher in this study, and 52.5% of patients had single brain metastases, 50% of patients had brain metastasis as the first site of distant metastasis, and 40% of patients had only central nervous system involvement. Considering the patients analyzed in this study, all underwent craniotomy, which indicated that the number of brain metastatic lesions was relatively small and suitable for surgical treatment, and therefore, this result was not remarkable. Another retrospective study enrolled 209 breast cancer patients with brain metastases who underwent craniotomy and showed that 121 (58%) patients had solitary brain metastases and 72 (34%) patients had only central nervous system involvement (22), which showed a similar trend compared to this study.

A multicenter, retrospective analysis of 219 breast cancer patients with brain metastases recently published by the American researchers showed that the inconsistency rates of ER, PR, and HER-2 between the primary lesions and brain metastatic lesions were 16.7% (36/216), 25.2% (53/210), and 10.4% (21/201), respectively, of which 36.3% (70/193) were inconsistent in the expression of anyone receptor (ER, PR, or HER-2), and the receptor conversion led to a subtype conversion of 22.8% (50/193) (23). These findings were also confirmed by the results of a subset analysis of brain metastases in a large meta-analysis, which explored the receptor discordance between the primary lesions and metastatic tumors, and the conversion rates of ER, PR, and HER-2 between the primary lesions and brain metastatic lesions were 20.8, 23.3, and 12.5%, respectively (11). This was the first study with the largest sample size to analyze the receptor discordance between the primary lesions and brain metastatic lesions in the Chinese patients, which showed similar results compared to that in the patients abroad. This study further described the conversion trend of ER, PR, and HER-2, in which PR in brain metastasis tended to change from positive to negative expression. Previous studies showed that ER and PR were more likely to be negative in the distant metastatic lesions compared to the primary lesions (11, 24–26). Nevertheless, the ratios of positive-to-negative and negative-to-positive in ER conversion were equal, and no obvious trends were found in this study. Therefore, further studies should be conducted to confirm this phenomenon related to the trend of conversion of receptor involvement. It will make important clinical implications if these observations are substantiated in further studies, which suggest that many patients do not receive appropriate systemic therapy for their metastases. However, if every patient with brain metastases undergoes neurosurgery to evaluate the immunohistochemical information of brain metastases, it will also bring a huge economic burden to patients and the medical insurance department. Since there is no prospective randomized study on the inconsistency of receptor expression between the primary lesions and brain metastatic lesions, the results of our study remain to be substantiated in future. So there is currently no guideline that clearly recommends that all patients with brain metastases should undergo surgery to evaluate recipient information.

A retrospective study enrolled 37 breast cancer patients with brain metastases found that the overall survival of patients without receptor conversion between the primary lesions and brain metastatic lesions was longer than that of patients with receptor conversion, and the overall survival for two groups was 31.1 and 19.1 months (*p* = 0.181), respectively (19). An article published by Niikura et al. in 2012 showed that in 182 patients with HER-2-positive primary breast cancer and systemic metastases, the HER-2 inconsistency was significantly associated with poorer survival (27), however, the paper published by Amir et al. reported that the HER-2 inconsistency had no deleterious effect on the overall survival of patients (12). These studies did not further analyze the inconsistency of HER-2 expression, which also indicated that the relationship between the differences in the expression levels of receptors and patients' survival was still inconclusive. Our study found that the inconsistency of HER-2 expression between the primary lesions and brain metastatic lesions was associated with better survival, and further analysis suggested that patients whose HER-2 expression was converted from positive in the primary lesions to negative in the brain metastatic lesions had a longer survival time. However, due to the small sample size and the lack of analysis of patients receiving treatment, the results of our study should be interpreted with considerable caution. Therefore, more randomized controlled trials with large sample sizes should be conducted to explore the correlation between receptor conversion and patients' survival in the future.

A retrospective study conducted by Lindstrom et al. showed that the HR inconsistency between the primary lesions and extracranial metastatic lesions was significantly associated with poor survival (28), however, the prospective trial conducted by Amir et al. suggested that the HR inconsistency between the primary lesions and extracranial metastatic had no adverse effect on the overall survival of patients (12). Another study explored the receptor expression in the breast cancer brain metastases, which suggested that loss or increase of HR expression in the brain metastatic lesions appeared to have no significant effect on patients' survival after diagnosis of BCBM (29). The results of our study suggested that patients with inconsistent HR expression between the primary lesions and brain metastatic lesions had better survival compared with those patients with consistent HR expression, in which the inconsistency rate of the ER expression in our study was 25.0%, which was higher than the rate from previous publications. Previous studies reported that loss of ER expression in the metastatic lesions was a negative prognostic factor for the survival of breast cancer patients (30–32), and a multicenter retrospective analysis of breast cancer patients with brain metastases showed that loss of ER expression in the brain metastatic lesions was associated with poor survival (*P* = 0.03), but the patients acquired ER expression simultaneously showed a survival advantage (23). Nevertheless, many studies on BCBM did not report these results (19, 33), therefore, further studies enrolling more patients should be conducted to substantiate these findings.

A study conducted by Caly et al. based on 257 breast cancer patients showed that the Ki-67 index is a prognostic factor for disease-free survival and overall survival (34). The cut-off value of the Ki-67 index was set to 40% in our study, and the breast cancer patients with brain metastasis whose Ki-67 of ≤ 40% in the primary lesions had a longer brain metastasis-free survival compared to those whose Ki-67 of >40% in the primary lesions. The cut-off value of the Ki-67 index of the brain metastatic lesions minus the Ki-67 index of the primary lesions was set at 10% in this study, and the difference in the survival time was observed between the two groups after diagnosis of brain metastases. Although we were still unable to determine whether the change in Ki-67 index between the primary lesions and brain lesions was related to patients' survival currently, which provided a direction for future studies.

This was the first study with the largest sample size to analyze the receptor expression in both primary lesions and brain metastatic lesions in the Chinese breast cancer patients, which was the main advantage of this study. We also conducted an exploratory analysis of the impact of receptor conversion on patients' survival. However, since it was a retrospective study with a relatively small sample size, the results of this study (especially the results related to survival) should be further substantiated by prospective studies with large sample sizes.

## Conclusion

In this study, 45.0% of breast cancer patients developed biomarker-conversion between the primary lesions and brain metastatic lesions, and the differences in the expression levels of the ER, PR, and HER-2, the change in Ki-67 index between the primary lesions and brain lesions may predict patients' survival.

## Data availability statement

The original contributions presented in the study are included in the article/Supplementary material, further inquiries can be directed to the corresponding author.

## Ethics statement

This study was reviewed and approved by the Ethics Committees of The Fifth Medical Center of PLA General Hospital. All patients and healthy individuals had given written informed consent.

## Author contributions

CJ wrote the main manuscript. ZJ, ZH, WXu, and WXi prepared Figures 1–8. ZS, TY, and JZ participated in writing manuspcript. All authors contributed to the article and approved the submitted version.

## Conflict of interest

The authors declare that the research was conducted in the absence of any commercial or financial relationships that could be construed as a potential conflict of interest.

## Publisher's note

All claims expressed in this article are solely those of the authors and do not necessarily represent those of their affiliated organizations, or those of the publisher, the editors and the reviewers. Any product that may be evaluated in this article, or claim that may be made by its manufacturer, is not guaranteed or endorsed by the publisher.
